# Performance of Body Adiposity Index and Relative Fat Mass in Predicting Bioelectric Impedance Analysis-Derived Body Fat Percentage: A Cross-Sectional Study among Patients with Type 2 Diabetes in the Ho Municipality, Ghana

**DOI:** 10.1155/2023/1500905

**Published:** 2023-04-17

**Authors:** Sylvester Yao Lokpo, Cephas Yao Ametefe, James Osei-Yeboah, William K. B. A. Owiredu, Linda Ahenkorah-Fondjo, Percival Delali Agordoh, Emmanuel Acheampong, Kwabena Obeng Duedu, Esther Ngozi Adejumo, Michael Appiah, Emmanuel Akomanin Asiamah, Emmanuel Ativi, Precious Kwablah Kwadzokpui

**Affiliations:** ^1^Department of Medical Laboratory Sciences, School of Allied Health Sciences, University of Health and Allied Sciences, Ho, Ghana; ^2^School of Public Health, Kwame Nkrumah University of Science and Technology, Kumasi, Ghana; ^3^Department of Molecular Medicine, School of Medicine and Dentistry, Kwame Nkrumah University of Science and Technology, Kumasi, Ghana; ^4^Department of Nutrition and Dietetics, School of Allied Health Sciences, University of Health and Allied Sciences, Ho, Ghana; ^5^Department of Biomedical Sciences, School of Basic and Biomedical Sciences, University of Health and Allied Sciences, Ho, Ghana; ^6^Department of Medical Laboratory Science, School of Public and Allied Health, Babcock University, Ilishan-Remo, Ogun State, Nigeria; ^7^Department of Medical Laboratory Sciences, Accra Technical University, Accra, Greater Accra Region, Ghana; ^8^Medical Laboratory Department, Ho Teaching Hospital, Ho, Ghana

## Abstract

**Objective:**

The study sought to determine the diagnostic accuracy of body adiposity index (BAI) and relative fat mass (RFM) to predict BIA-derived BFP among patients with type 2 diabetes in the Ho municipality. *Materials and Method*. This hospital-based cross-sectional study involved 236 patients with type 2 diabetes. Demographic data, including age and gender were obtained. Height, waist circumference (WC), and hip circumference (HC) were measured using standard methods. BFP was estimated on a bioelectrical impedance analysis (BIA) scale. The validity of BAI and RFM as alternative estimates for BIA-derived BFP was evaluated based on mean absolute percentage error (MAPE), Passing-Bablok regression, Bland-Altman plots, receiver-operating characteristic curve (ROC), and kappa statistics analyses. A *p* value less than 0.05 was considered statistically significant.

**Results:**

BAI showed systematic bias in estimating BIA-derived BFP in both genders, but this was not evident between RFM and BFP among females (*t* = −0.62; *p* = 0.534). While BAI showed “good” predictive accuracy in both genders, RFM exhibited “high” predictive accuracy for BFP (MAPE: 7.13%; 95% CI: 6.27-8.78) among females according to MAPE analysis. From the Bland-Altman plot analysis, the mean difference between RFM and BFP was acceptable among females [0.3 (95% LOA: -10.9 to 11.5)], but both BAI and RFM recorded large limits of agreement and low Lin's concordance correlation coefficient with BFP (Pc < 0.90) in the two gender populations. The optimal cut-off, sensitivity, specificity, and Youden index for RFM were >27.2, 75%, 93.75%, and 0.69, respectively, while those of BAI were >25.65, 80%, 84.37%, and 0.64, respectively, among males. Among females, the values for RFM were >27.26, 92.57%, 72.73%, and 0.65, whereas those of BAI were >29.4, 90.74%, 70.83%, and 0.62, respectively. The accuracy of discriminating between BFP levels was higher among females [BAI (AUC: 0.93) and RFM (AUC: 0.90)] compared to males [BAI (AUC: 0.86) and RFM (AUC: 0.88)].

**Conclusion:**

RFM had a better predictive accuracy of BIA-derived BFP in females. However, both RFM and BAI failed as valid estimates for BFP. Furthermore, gender-specific performance in the discrimination of BFP levels for RFM and BAI was observed.

## 1. Introduction

Obesity has become a major public health problem, as its prevalence has increased around the world. By 2025, global projections for men and women are expected to reach 18% and 21%, respectively [1]. Obesity represents an imbalance in energy intake and expenditure, thus increasing the amount of adipose tissue and activating the endocrine systems [2]. The resulting increase in adipokines influences metabolism and thus manifests changes in appetite, satiety, energy expenditure, insulin sensitivity and secretion, glucose and lipid metabolism, fat distribution, and neuroendocrine modulation, as well as immune system function [3]. Therefore, obesity mediates the development of cardiometabolic complications such as hypertension, type 2 diabetes, dyslipidaemia [4], and some malignancies [5].

Globally, the body mass index (BMI) is used as a surrogate metric for determining obesity. BMI is a weight-for-height calculation that takes into account fat mass, fat-free mass, and body fluid [6]. It is calculated as body weight in kilogram divided by height in meters squared (kg/m^2^). Due to the relative ease of its calculation, BMI is widely used as a measure of weight status in epidemiology, clinical care, and clinical nutrition. Furthermore, BMI values greater than 25 kg/m^2^ [classified as high and interpreted as being overweight or obese when BMI ≥ 30 kg/m^2^] have been found to be a significant predictor of all-cause death [7]. However, the BMI cannot differentiate between lean and fat mass; hence, such levels of body adiposity can only be quantified using other distinct methodologies to achieve individualized health care for patients [8]. For example, body fat percentage (BFP), which is defined as the percentage of total body weight, that is fat, is considered a credible indicator of body adiposity due to its association with metabolic complications regardless of body weight [7].

To compensate for the ineffectiveness of BMI, several basic anthropometric indicators, including body adiposity index (BAI) and relative fat mass (RFM), have been proposed [9, 10]. The RFM equation is a straightforward approach for estimating BFP based on height and waist circumference (WC). Woolcott and Bergman [10] created and validated the RFM using data from the National Health Examination Survey (NHANES). Also, BFP is estimated using the body adiposity index (BAI). It is a straightforward calculation that takes into account the hip circumference (HC) and body height. The BAI performed well during its validation in a population different from the one with which it was designed [9]. Therefore, Bergman et al. [9] argued that it would not require additional changes for factors such as gender and age to improve its performance. Hence, the BAI was touted as a quick, low-cost, and noninvasive evaluation tool for use in clinical practice.

Meanwhile, various high-performance reference techniques have emerged for measuring body fat, including hydrostatic weighing, dual-energy X-ray absorptiometry, total body potassium, air displacement plethysmography, and isotope dilution. Unfortunately, these methods are costly and mostly limited to laboratory settings and therefore may not be appropriate for epidemiological investigations [11]. Bioelectrical impedance analysis (BIA) is a relatively cheaper method of estimating body fat, but has not gained widespread use in primary healthcare settings in Ghana, including the present study site, probably due to cost. Given that healthcare professionals continue to rely on simple anthropometric indices to assess total body fat, it is important to evaluate their performance against methods that had shown high performance against the reference techniques [12, 13].

Furthermore, contrary to the view that BAI performs well in different populations [9], findings from previous studies have suggested otherwise. While Thivel et al. [14] observed a modest correlation between BAI and dual-energy X-ray absorptiometry among adolescents aged 12–16 years, Chang et al. [15] found that BAI had the propensity to overestimate and underestimate BFP in men and women aged 55 to 96 years measured by dual-energy X-ray absorptiometry at 15% and 40%, respectively. Likewise, Guzmán-León et al. [16] and more recently Senkus et al. [17] found that RFM is a valid estimate of total body fat whereas Encarnação et al. [18] had reported findings to the contrary. Therefore, the discrepancies, restrictions, and disputes about the validity of basic anthropometric indices, along with the lack of information among some groups with chronic diseases, suggest a knowledge gap that requires further research in this area. In view of the foregoing, we devised the present study to evaluate the accuracy of BAI and RFM in predicting BFP measured by BIA in people with type 2 diabetes in the Volta Region of Ghana.

## 2. Materials and Methods

### 2.1. Study Design and Study Site

This study used a hospital-based cross-sectional study design. The study was carried out at the Diabetic Clinic of the Ho Municipal Hospital located in Ho, the capital of the Volta Region of Ghana. Ho is located between Mount Adaklu and Mount Galenukui or the Togo Atakora Range. The city used to serve as the administrative capital of British Togoland before transitioning into being a part of the present-day Volta Region. The recent Ghana Population and Housing Census for 2021 estimates the population of the Ho municipality at 180,420. This represents 10.9% of all people in the Volta Region, Ghana. Females constitute 52.9%, and males represent 47.1 percent. The municipality is bordered on the south by the Adaklu and Agotime Ziope Districts, on the north and west by the Ho West District, and on the east by the Republic of Togo.

### 2.2. Study Population and Sampling Technique

Patients with type 2 diabetes aged 20 and older receiving care in the Diabetic Clinic of Ho Municipal Hospital at the time of this study, those who were able to take anthropometric measures, and also volunteered to participate in this study were conveniently recruited into the study. However, patients under the age of 20, patients with type 1 diabetes, had difficulty moving, and those who refused to participate in the study were excluded.

### 2.3. Sample Size Determination

Using the Raosoft online calculator (http://www.raosoft.com), we computed a minimum recommended sample size of 197 from a population of 400 type 2 diabetic patients who regularly attend the diabetic clinic at 95% confidence interval with 5% margin of error. However, this study recruited a total of 236 individuals with type 2 diabetes.

### 2.4. Data Collection

#### 2.4.1. Demographic Data and Anthropometric Evaluation

A trained final year health student from the University of Health and Allied Sciences collected the data. A semistructured questionnaire was used to collect demographic information (age and sex). All measures were taken after the research participants had fasted overnight (10-12 hours). A stadiometer was used to measure the height to the closest 0.1 cm as they stood erect, straight back, the heels together, and the feet slightly spread. The bioelectric impedance analysis (BIA) body composition device (Omron BF-511; Omron Healthcare Co., Ltd., Kiyoto, Japan) was used to obtain other anthropometric indices such as weight, BMI, and BFP. The device operates on the principle of resistance and reactance to bodily tissues when a tiny electrical current is injected into the body to assess adiposity [19]. It employs eight electrodes in a tetrapolar configuration, with the participant stepping barefoot onto the scale, gripping the display unit with both hands, and extending the arms parallel to the floor while standing erect. The participant's information on age, height (cm), and gender was entered into the device prior to mounting the scale. After weight was measured directly by the device, the BMI and BFP values were generated. Waist circumference (WC) was measured in centimeters at the midpoint between the lower border of the rib cage and the iliac crest, at the midaxillary line, with patients standing erect and breathing normally. Hip circumference (HC) was measured in centimeters as the maximum circumference around the buttocks at the largest width of the gluteal protuberance. The waist-hip ratio (WHR) was calculated as WC (cm) divided by HC (cm). BAI for estimating body fat was calculated based on the formula BAI = [hip (cm)/height (m) 1.5] − 18 [9] while RFM estimation of body fat was calculated using the equation RFM = 64 − (20 × height/waist circumference) + (12 × sex); sex = 0 for men and 1 for women [10].

#### 2.4.2. Definition of Obesity Based on BFP, RFM, and BAI Algorithms

For BFP using BIA, obesity was defined as BFP < 25% for men and ≥35% for women [20]. For RFM, obesity was defined as RFM ≥ 25% for men and ≥35% for women [21]. For BAI, age- and gender-specific obesity was defined as, for males: >26 (20-39 years), >27 (40-59 years), and >29 (>59 years); and for females: >38 (20-39 years), >39 (40-59 years), and >41 (>59 years) [22].

### 2.5. Statistical Analysis

Data were collected and entered into Microsoft Office Excel 2016 spreadsheet for cleaning. The normality test was performed on all continuous variables. Continuous variables were expressed as means and ± standard deviation of the mean, while categorical variables were expressed as frequencies and corresponding proportions. Comparisons of continuous variables between the two gender categories were performed using unpaired Student's *t*-test, while group comparisons of categorical variables were performed using chi-square or Fisher's exact test statistic. Lin's concordance correlation coefficients were estimated to determine how well RFM and BAI compare with BIA. Passing-Bablok regression and Bland-Altman plots of the differences in mean analyses were performed to determine the bias of BAI and RFM in predicting BFP. The mean absolute percentage error (MAPE) was used to assess the predictive accuracy between the three diagnostic instruments. Moreover, the diagnostic accuracy was evaluated based on optimal cut-off, sensitivity, and specificity analyses. The area under the curve (AUC) analysis of the receiver-operating characteristic curves (ROC) was used to determine the discriminatory ability of the obesity indices. The level of agreement between obesity indices and BFP were evaluated based on unweighted kappa coefficient (*k*) using the interrater agreement analysis. Linear regression models were used to determine the relationship of BFP with BAI and RFM. A *p* value of <0.05 was considered statistically significant. IBM Statistical Package for Social Sciences version 22.00 (SPSS Inc., Chicago, USA) (http://www.spss.com) and MedCalc version 12.3.2 for windows (MedCalc software bvba, Acacialaan 22, B-8400 Ostend, Belgium) (http://www.medcalc.org) were used for data analysis.

### 2.6. Ethical Consideration

Ethical clearance was obtained from the University of Health and Allied Sciences Research Ethics committee (UHAS-REC) with protocol identification number: UHAS-REC A.10 (17) 20-21. The study was approved by the management of Ho Municipal Hospital. Signed informed consent forms were obtained from each participant. The electronic data file was protected until it was utilized for analysis, ensuring data confidentiality.

## 3. Results

### 3.1. Baseline Characteristics and Prevalence of Obesity Indices among Study Participants

A total of 236 participants, comprising 132 females and 104 males were recruited into this study. The average age of study participants was 51.0 ± 8.7 years with more than 70% within the 40- and 59-year category. Generally, the average anthropometric indices (BMI, WC, and HC) of female participants were statistically higher than those observed in their male counterparts except for height where the reverse was the case. Obesity defined using BFP, BAI, and RFM criteria in the total study population was 148 (62.7%), 73 (30.9%), and 165 (69.9%), respectively. There were significant gender disparities in obesity indices, with female participants recording higher proportions compared to male participants [BFP: 108 (81.8%) vs. 40 (34.5%)] and [RFM: 116 (87.9%) vs. 49 (47.1%)]. Obesity defined by the BAI criterion revealed statistically comparable proportions between male and female participants (see Table 1).

Compared to BIA-derived BFP reference method, BAI produced a significantly lower body fat estimate among females (41.27 ± 8.10 vs. 34.40 ± 6.36) but significantly higher body fat estimates among males (22.68 ± 9.82 vs. 25.65 ± 6.02) with a mean difference of 6.87 (p < 0.001). Though not significant, RFM yielded a higher body fat estimate compared to BIA (mean diff = −0.32, p = 0.534) among women. Among males, RFM produced significantly higher body fat estimates compared to BIA (mean diff = −2.41; p = 0.001). RFM was identified to have a high predictive accuracy for BIA-derived BFP among females with a mean absolute percentage error of 7.13%. Both BAI and RFM had good prediction of BFP among males with MAPEs of 15.73% and 18.58%, respectively (Table 2).

**Figure 1:**
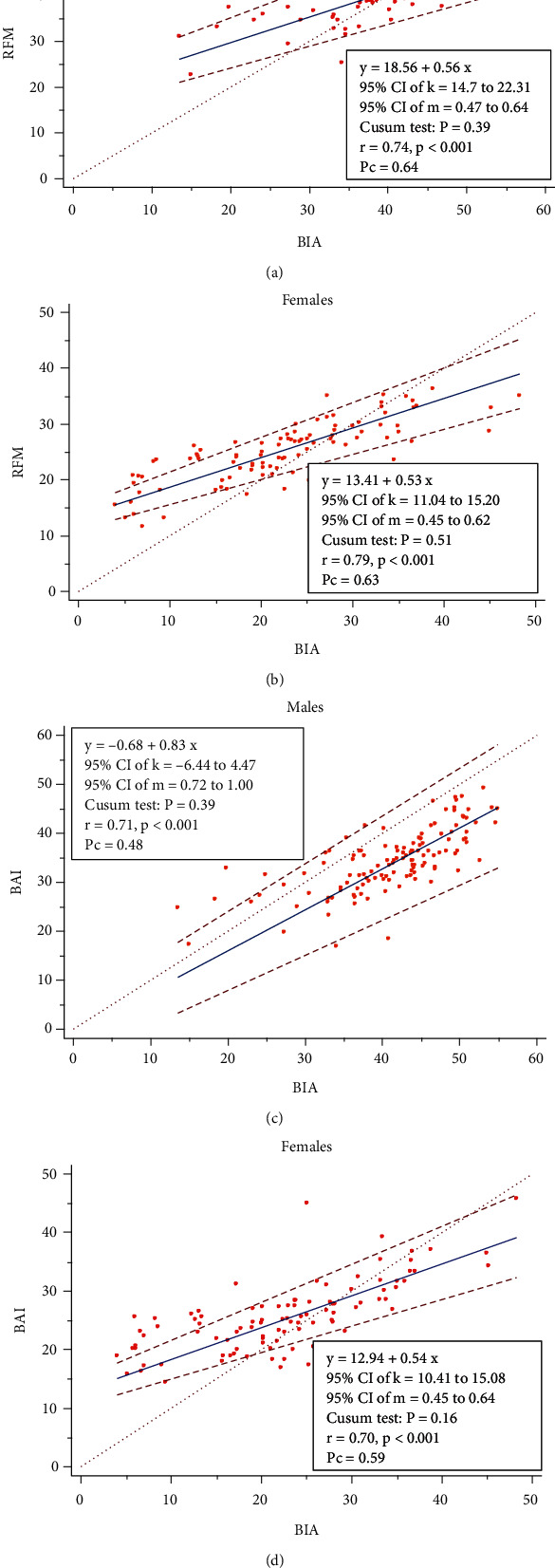
Relationship of BAI and RFM with BFP (via BIA) stratified by gender presented using Passing-Bablok regression plot. *k*: intercept, *m*: slope, *r*: Pearson correlation coefficient, Pc: Lin's concorrdance correlation coefficient, %BF: percentage body fat using BIA (bioelectrical impedance analysis). Pc < 0.90 rated poor agreement, Pc 0.90 - 0.95 moderate agreement, Pc 0.95-0.99 substantial agreement, and Pc > 0.99 excellent agreement.

Figure 1 shows the correlations of BAI and RFM with BFP. There was a significantly strong positive correlation between BAI or RFM and BFP measured using BIA (*p* < 0.001). Using Passing-Bablok regression analysis, BAI was comparable with BIA in estimating BFP among females (intercept = −0.68; slope = 0.83). For men, BIA showed high systematic and proportional differences between BAI and BIA in estimating BFP (intercept = 12.94; slope = 0.54). The ratings of the strength of agreement was based on those proposed by McBride with LCCC < 0.90 rated as poor, 0.90 to 0.95 as moderate, 0.95 to 0.99 as substantial, and >0.99 as almost perfect agreement. Therefore, the concordance between BAI and BIA was 0.48 and 0.59 among women and males, respectively. Additionally, there were proportional and systematic differences between RFM and BIA among men (intercept = 18.56; slope = 0.55) and women (intercept = 13.40; slope = 0.53) based on Passing-Bablok regression analysis. The concordance between RFM and BIA was 0.64 for women and 0.63 for men. Cusum linearity test did not indicate significant deviation from linearity for both BAI and RFM estimates in men or females (*p* > 0.05).

**Figure 2:**
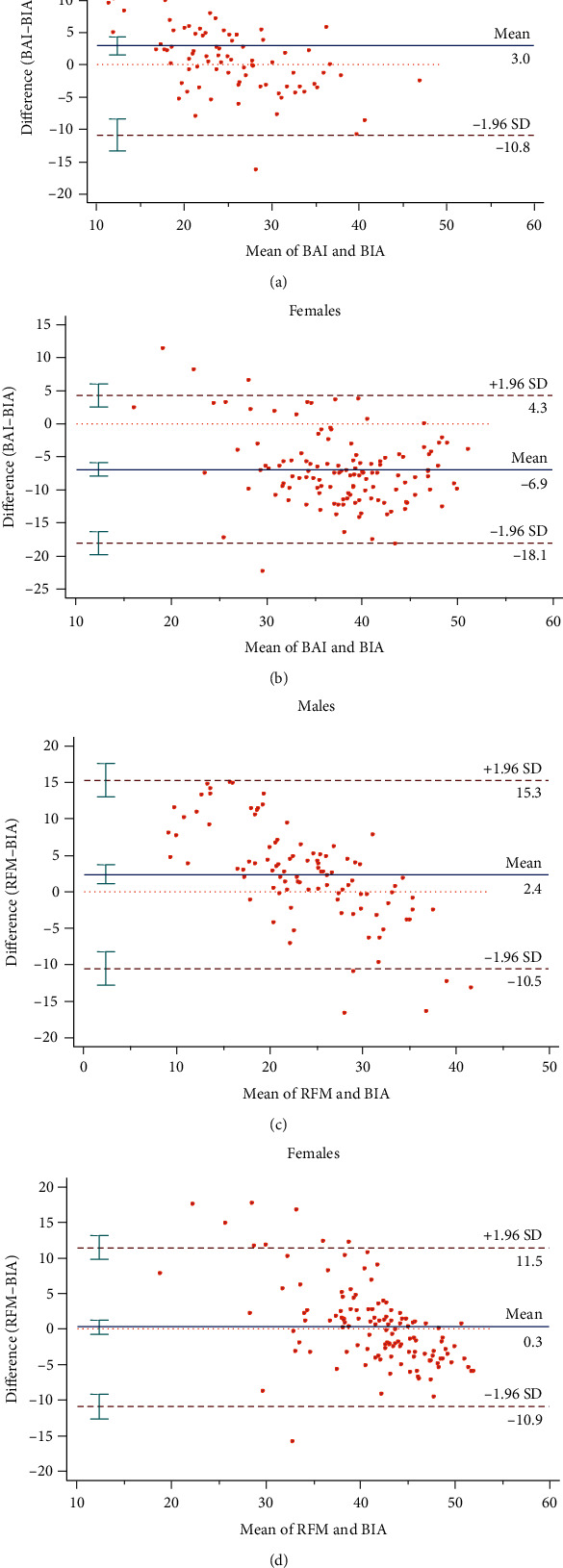
Bland-Altman's plot of mean difference between BIA determined BFP and BAI or RFM for males and females.

In the Bland-Altman graphs of mean differences in Figure 2, there was acceptable agreement between RFM and BIA in the estimation of BFP among women (mean diff = 0.3; LOA = −10.9–11.5) but that level of agreement was not observed among men. BAI overestimated BIA among males (mean diff = 3.0; LOA = −0.8–16.8) and underestimated BIA among females (mean diff = −6.9; 95%LOA = −18.1–4.3).

**Figure 3:**
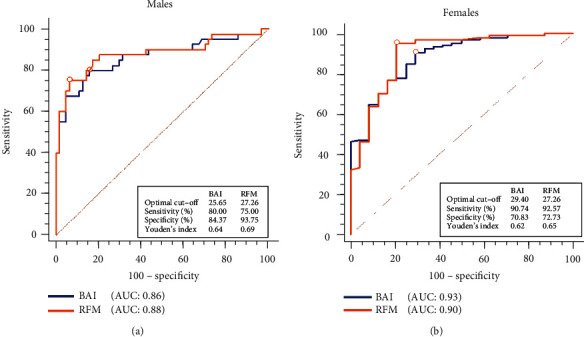
Receiver-operating characteristic (ROC) curves of BAI and RFM in diagnosing obesity using BIA determined body fat percentage as reference classification. Marked point corresponds to Youden's index.

In Figure 3, an optimal diagnostic cut-off value of 25.65 was determined for BAI in the diagnosis of obesity with a sensitivity of 80% and a specificity of 84.37%, while a RFM cut-off value of 27.26 with sensitivity and specificity of 75% and 93.75%, respectively, was observed among males. Among females, an optimal cutoff value of 29.4 was determined to classify obesity using BAI (sensitivity = 90.74%, specificity = 70.73%) while for RFM, a threshold value of 27.26 produced sensitivity and specificity of 92.57% and 72.75%, respectively. A maximum Youden index of 0.65 and 0.69 with RFM was observed at a diagnostic cut-off of 27.26 among females and males, respectively. For BAI, Youden indices of 0.62 and 0.64 were produced for the cut-off values of 29.4 and 25.65 among females and males, respectively. Areas under the ROC curve of 0.88 and 0.90 for RFM and 0.86 and 0.88 for BAI were recorded among males and females, respectively.

As presented in Table 3, there was a “good” agreement between RFM and BIA in the determination of obesity states (*k* = 0.69). The BAI agreement ranged from “fair” with BIA to “good” with RFM in the diagnosis of obesity. Furthermore, BAI was “fairly” correlated with RFM in the diagnosis of obesity (*k* = 0.25).

## 4. Discussion

Worldwide, obesity and type 2 diabetes constitute the major public health issues in modern societies, and are said to frequently coexist, with statistics showing that approximately 60-90% of all patients with type 2 diabetes are obese or at high risk of developing obesity [23]. However, an accurate diagnosis of body fat composition requires expensive equipment and specialised skills and, in certain situations, exposure to potentially hazardous radiations [24]. Therefore, the search for alternative diagnostic procedures that perform well, and are safe, and reasonably simple to use becomes critical, especially in low-resource settings. To our knowledge, however, no study had assessed the ability of simple anthropometric indices to accurately predict BIA-derived body fat composition measures among a high-risk population of type 2 diabetes in the Volta Region. Therefore, the objective of the present study was to evaluate the ability of RFM and BAI to predict BFP estimated by BIA among patients with type 2 diabetes in the Ho municipality.

In this study, we found that BAI demonstrated systematic bias in estimating BFP measured using BIA as evidenced by the significant mean differences between them in both genders while the difference in the mean RFM and BFP was not significant among females (*t* = −0.62; *p* = 0.534) indicating nonexisting bias. Furthermore, while BAI showed “good” predictive accuracy in both genders, RFM exhibited “high” predictive accuracy for BFP among females according to the MAPE analysis (MAPE: 7.13%; 95% CI: 6.27-8.78) (Table 2). The performance of RFM among females is consistent with Woolcott and Bergman [10] who in the validation study demonstrated that RFM was more accurate and had fewer errors in defining body fat than BMI among females. RFM is an emerging estimator of total BFP based on WC, height, and biological sex [10], and its development was intended to overcome gender-related issues in the BAI method, as well as BMI limitations [17] using heterogeneous study populations that included Mexican-Americans, European-Americans, and African-Americans. Thus, the difference observed in the ability of RFM to estimate body fat with respect to gender is an indication that RFM may not demonstrate same predictive accuracy at the subpopulation level.

Based on Bland-Altman plot analysis, we observed that BAI overestimated body fat in individuals with lower BFP (males) and underestimated body fat in individuals with higher BFP (females) (Figures 2(a) and 2(b)). However, RFM overestimated body fat in both males and females (Figures 2(c) and 2(d)). The findings in relation to BAI correspond to a study among Brazilians with type 1 diabetes in which BAI had the tendency to overestimate adiposity in boys and underestimate adiposity in girls using the dual-energy X-ray absorptiometry method as reference standard [25]. Fedewa et al. [26] advanced these findings by demonstrating that BAI overestimates BFP in men without Down syndrome, but underestimated BFP in women without Down syndrome compared with dual-energy X-ray absorptiometry method. Furthermore, in addition to exhibiting wider limits of agreement (LOA), BAI underestimated BFP by a mean bias of 6.4% among females [-6.4 (95% LOA: -18.1 to 4.3)] and overestimated BFP by a mean bias of 3.0% among males [3.0 (95% LOA: -10.8 to 16.8)] (Figures 2(a) and 2(b)). The inconsistency of BAI in estimating the true value of BIA-derived BFP among the gender populations reduces its ability to accurately predict total body fat at the subpopulation level. According to Chang et al. [15], differences in the distribution of body fat in the gluteal–femoral region and abdominal area in women and men, respectively, make BAI ineffective as it overlooks the abdominal fat depot and uses HC and height to predict body fat. Moreover, previous studies have shown that BAI overestimates the value of body fat in people with BFP ≤ 25% and underestimates the true value of body fat in individuals with BFP more than 30% [9, 15, 27]. Therefore, it was argued that BAI would perform best at values between 20 and 30%, which is exactly the lowest health risk generally classified for BFP as appropriate [28, 29].

In contrast, RFM was better at estimating the true value of BFP in both males and females, in addition to demonstrating tighter limits of agreement. This probably underscores the superior agreement between the RFM and BIA in defining BFP in both gender populations, which agreement was found to be more profound among females [0.3 (95% LOA: -10.9 to 11.5)] compared to males [2.4 (95% LOA: -10.5 to 15.3)] (Figures 2(c) and 2(d)). However, though the strength of agreement between RFM and BFP among females could be regarded as acceptable, both BAI and RFM failed as valid estimates of BFP owing to the large differences in the limits of agreement. For an alternative method of measurement to be valid, aside from the closeness of its value to the true estimate of the reference method, the limits of agreement must also be as tight as possible. However, our findings agree with Paek et al. [21] where RFM exhibited similar performance in estimating the true value of BFP and the limits of agreement when evaluated against the dual-energy X-ray absorptiometry in both genders among a Korean adult population.

Another interesting finding is the observation that, though BAI and RFM achieved strong correlations with BIA-derived BFP, both indices demonstrated poor concordance according to the LCCC analysis (with Pc ranging from 0.48 to 0.64) (Figures 1(a)–1(d)). The strong linear relationship of BAI and RFM with BFP is consistent with Encarnação et al. [18] and Paek et al. [21], but the poor concordance with BIA-derived BFP is also in tandem with results obtained by Appelhans et al. [30], Cerqueira et al. [27], and Ribeiro da Costa et al. [31]. However, it is argued that when two methods of measurement achieve strong correlation, this does not necessarily translate into a perfect agreement between the two methods. Hence, beyond estimating the correlation coefficient, which is a measure of the closeness of data about the line of best fit, the LCCC, on the other hand, measures how far or close that line is from the 45-degree line through the origin, indicating the degree of agreement between the two methods [32]. Hence, the failure of BAI and RFM to meet the minimum threshold of LCCC (Pc = 0.90) according to our data calls into question their reliability as valid estimates of BFP. However, based on the interrater reliability (agreement) analysis, we found a “good” agreement between RFM and BIA (*k* = 0.69) and a “fair” agreement between BAI and BIA (*k* = 0.25) to define BFP among the total study respondents (Table 3). This resonates with the results of Corrêa and colleagues [33] who found a higher degree of agreement between RFM and BIA (*k* = 0.67) compared to BMI and BIA (*k* = 0.60) among Brazilian adults.

Finally, from the ROC curve analysis, both BAI and RFM appear to demonstrate higher accuracy in discriminating between BFP levels among females compared to males. While BAI performed better in females [BAI cut‐off > 29.4; AUC: 0.93 and RFM cut‐off > 27.26; AUC: 0.90], RFM demonstrated a better accuracy in males [BAI cut‐off > 25.65; AUC: 0.86 and RFM cut‐off > 27.2; AUC: 0.88] (Figures 3(a) and 3(b)). In contrast, Woolcott and Bergman [10] found that RFM was superior in the diagnosis of BFP (AUC < 0.93) compared to BMI in both genders. Essentially, our study differs in population type (patients with type 2 diabetes) and reference method (BIA) from the Woolcott and Bergman [10] study who utilized data generated from the heterogeneous NAHNESS cohort using the dual-energy X-ray absorptiometry as the reference method. Additionally, the Youden index which reflects the overall diagnostic performance of adiposity indices evaluated in this study was higher for RFM compared to BAI in both genders (Figures 3(a) and 3(b)).

However, some inherent limitations are acknowledged so caution should be exercised in the interpretation of our study findings. This is a single-center hospital-based study so generalization of the study findings is not possible. Furthermore, we did not take into account the nutritional status of study participants, as this is likely to influence our findings. Moreover, while we acknowledge the limitations associated with the use of BIA to estimate body fat, it is also worth mentioning that BIA-derived body fat estimates have been found to be valid estimates when evaluated against the more sensitive methods including, e.g., air displacement plethysmography and dual-energy X-ray absorptiometry, thus considered a valid method in research and population samples [12, 13]. Hence, the present study provides important baseline information that could guide future studies in the study area.

## 5. Conclusion

RFM had a better predictive accuracy of BIA-derived BFP in females. However, both RFM and BAI failed as valid estimates for BFP. Furthermore, gender-specific performance in the discrimination of BFP levels for RFM and BAI was observed.

## Figures and Tables

**Table 1:** Baseline characteristics and prevalence of obesity indices among study participants.

Parameter	Total (*n* = 236)	Females (*n* = 132)	Males (*n* = 104)	*p* value
Age (years)	51.0 ± 8.7	52.2 ± 8.2	49.4 ± 9.0	**0.013**
Age category (years)				
20-39	27 (11.4)	9 (6.8)	18 (17.3)	**0.0379**
40-59	187 (79.2)	109 (82.6)	78 (75.0)	
≥60	22 (9.3)	14 (10.6)	8 (7.7)	
Weight (kg)	73.6 ± 15.6	73.3 ± 15.5	74.1 ± 15.8	0.6966
Height (cm)	163.2 ± 11.4	157.5 ± 6.6	170.3 ± 12.2	**<0.0001**
BMI (kg/m^2^)	27.6 ± 5.9	27.6 ± 5.9	25.3 ± 5.0	**<0.0001**
WC (cm)	90.8 ± 15.8	93.5 ± 15.3	87.3 ± 15.9	**0.0027**
HC (cm)	99.6 ± 16.6	104.2 ± 14.9	93.8 ± 17.0	**<0.0001**
BFP	32.9 ± 13.1	41.3 ± 8.4	22.3 ± 9.9	**<0.0001**
Obese (%)	148 (62.7)	108 (81.8)	40 (38.5)	**<0.0001**
Nonobese (%)	88 (37.29)	24 (18.18)	64 (61.5)	
BAI	30.25 ± 9.64	34.85 ± 8.31	24.42 ± 7.9	**<0.0001**
Obese (%)	73 (30.9)	36 (27.3)	37 (35.6)	0.2021
Nonobese (%)	163 (69.1)	96 (72.7)	67 (64.4)	
RFM	33.3 ± 13.1	41.03 ± 9.02	23.4 ± 10.6	**<0.0001**
Obese (%)	165 (69.9)	116 (87.9)	49 (47.1)	**<0.0001**
Nonobese (%)	71 (30.1)	16 (12.1)	55 (52.9)	

Data presented as mean ± standard deviation and frequency with proportion in parenthesis. BMI: body mass index; BAI: body adiposity index; RFM: relative fat mass; BFP: body fat percentage; HC: hip circumference; WC: waist circumference. The p value is significant at <0.05.

**Table 2:** Pairwise difference and MAPE in body fat estimates using BAI, RFM, and BIA among participants.

	BAI	RFM	^∗^BIA	MD	T	*p* value	MAPE (95% CI)
Female	34.40 ± 6.36		41.27 ± 8.10	6.87	13.53	<0.001	18.25% (16.70-21.31)
		41.59 ± 5.02	41.27 ± 8.10	-0.32	-0.62	0.534	7.13% (6.27-8.78)
	34.40 ± 6.36	41.59 ± 5.02		7.2	27.42	<0.001	23.18% (21.03-25.42)

Male	25.65 ± 6.02		22.68 ± 9.82	-2.96	-4.16	0.001	15.73% (12.47-20.84)
		25.09 ± 5.43	22.68 ± 9.82	-2.41	-3.62	0.001	18.58% (13.45-22.64)
	25.65 ± 6.02	25.09 ± 5.43		-0.55	-1.52	0.132	7.45% (6.05–8.66)

Data presented as mean ± standard deviation; BAI: body adiposity index; RFM: relative fat mass; BIA: bioelectrical impedance analysis; MAPE: mean absolute percentage error; ^∗^reference method. MAPE < 10% = high, 10%-20% = good, 20%-50% = reasonable, >50% = poor. ^∗^Reference test method.

**Table 3:** Interrater reliability (agreement) analysis of various methods (BIA, BAI, and RFM) in diagnosing obesity.

Test	^∗^BIA		BAI	
	Kappa (K)	95% CI	Kappa (K)	95% CI
RFM	0.689	0.592-0.786	0.250	0.167-0.332
BAI	0.266	0.171-0.361		

BMI: body mass index; BAI: body adiposity index; RFM: relative fat mass; BIA: bioelectrical impedance analysis. Kappa < 0.20 = poor, 0.21-0.40 = fair, 0.41-0.60 = moderate, 0.61-0.80 = good, and 0.81-1.00 = very good. ^∗^Reference test method.

## Data Availability

The data used for this study is available from the corresponding author on reasonable request.

## Abbreviations

WHO: World Health Organization
NCDS: Noncommunicable diseases
GDHS: Ghana Demographics Health Survey
NHANES: National Health Examination Survey
DXA: Dual-energy X-ray absorption
BMI: Body mass index
BAI: Body adiposity index
RFM: Relative fat mass
BFP: Body fat percentage
WC: Waist circumference
HC: Hip circumference
WHtR: Waist-height ratio
WHR: Waist-hip ratio
CAT: Computerized axial tomography
CI: Confidence interval
BIA: Bioelectrical impedance analysis.
